# Hepatocellular carcinoma in Pacific Islanders: comparison of Pacific Island-born *vs*. US-born

**DOI:** 10.20517/2394-5079.2022.85

**Published:** 2023-03-17

**Authors:** Shelby K. Yee, Brenda Y. Hernandez, Sandi Kwee, Linda L. Wong

**Affiliations:** 1 Department of Surgery, John A. Burns School of Medicine, Honolulu, HI 96813, USA.; 2 University of Hawaii Cancer Center, Honolulu, HI 96813, USA.

**Keywords:** hepatocellular carcinoma, Pacific Islander, nativity, risk factors, liver resection, liver transplant

## Abstract

**Aim:**

To describe demographic, clinical, and outcome differences in Pacific Island-born (PI-born) compared to US-born hepatocellular carcinoma (HCC) patients of Pacific Island ancestry within a clinical cohort in Hawaii.

**Methods:**

A prospectively collected database of 1608 patients diagnosed with HCC over a 30-year period (1993–2022) identified 252 patients of Pacific Islander ethnicity. Data collected: demographics, medical history, laboratory data, tumor characteristics, treatment, and survival. Patients were divided into two groups: PI-born and US-born. Categorical variables were analyzed using ANOVA and chi-square analysis. Odds ratios with 95% confidence intervals were calculated using univariate and multivariate logistic regression. Overall survival was evaluated using Kaplan-Meier analysis.

**Results:**

PI-born patients were younger (57.3 *vs*. 61.8 years, *P* = 0.002) and more likely to have hepatitis B (OR 14.10, 7.50–26.50) and underlying cirrhosis (OR 2.28, 1.17–4.45). In comparison, US-born patients had a significantly higher likelihood of Hepatitis C, nonalcoholic steatohepatitis/nonalcoholic fatty liver disease, history of non-HCC cancer, and positive smoking history compared to PI-born patients. PI-born patients were more likely to forego treatment (OR 3.22, 1.77–5.87) and be lost to follow-up (OR 9.21, 1.97–43.03). Both groups were equally likely to have the opportunity for curative surgical treatment (liver resection or transplant). US-born status was associated with higher mortality risk, while transplantation was associated with lower mortality risk. The PI-born cohort demonstrated higher overall survival at 3 and 5 years compared to US-born.

**Conclusion:**

HBV remains the primary risk factor for HCC in PI-born patients, whereas HCC in US-born patients is more associated with the adoption of a Westernized lifestyle.

## INTRODUCTION

Hepatocellular carcinoma (HCC) is both the sixth most common cancer and the sixth leading cause of cancer mortality worldwide^[1]^. Historically, viral hepatitis has been the primary risk factor for HCC and accounted for 50% of all cases, but obesity and metabolic disorder-related liver disease are responsible for the ongoing disease burden^[2–4]^. Disparities in incidence/prevalence, diagnosis, treatment, and outcomes of primary liver cancer in different populations have been recognized and may depend on factors including race/ethnicity, gender, and socioeconomic status^[5–9]^.

Pacific Islanders have one of the highest incidences of HCC, primarily attributed to the high prevalence of chronic hepatitis B (HBV)^[10,11]^. Despite attempts at prevention and delay of HCC with vaccination and antiviral agents, significant population disparities persist, likely due to differences in access to general healthcare and decreased utilization of treatment modalities^[12,13]^. While previous studies have described patterns and trends of HCC in Pacific Islanders, accurate conclusions have been difficult to draw as data is often reported together with Asian American patients, who are a distinct demographic group.

No previous studies have explored the differences in Pacific Islander patients based on birthplace. Compared to US-born Pacific Islander patients, patients born in the Pacific Islands may have different exposures and risk factors for developing disease and may face challenges associated with immigration. The Pacific Island countries and territories encompass thousands of islands that range economically from highly urban to largely unsettled and rural. Most are classified as low-middle income countries, with up to 25% of the population living with incomes below the poverty line. The health infrastructure remains severely underdeveloped in most Pacific Island regions. Rapid affordable laboratory diagnostics, histopathology, and radiology services are unavailable in many regions, and accessibility to treatment remains low; thus, many patients in these regions seek tertiary-level care in neighboring countries, including the United States (often in Hawaii)^[14]^. However, language barriers, low educational attainment, poverty, low level of urbanization, and lack of insurance can severely limit patients’ abilities to access healthcare resources^[15–18]^.

Hawaii is the state with the largest Native Hawaiian/Pacific Islander population and is also the state with the third highest incidence of HCC in the United States^[17,19]^. With a target patient population that spans multiple generations, it is an ideal location to study the underlying risk factors and trends in the development of HCC in Pacific Islanders. This study uses a well annotated database of patients with liver cancer in Hawaii over three decades to identify and describe differences within the Pacific Islander population based on nativity.

## METHODS

This is a retrospective cohort study of 1608 HCC cases referred over a 30-year period (1993–2022) to a surgical practice associated with the only liver transplant center and center for liver disease in Hawaii (initially located at St. Francis Medical Center/Hawaii Medical Center-East, then Queens Medical Center since 2012), which manages ~60%−70% of the HCC cases in the state. This center is also the tertiary referral center for the United States Pacific territories (including the United States territories of American Samoa, Guam, and the Northern Mariana Islands) as well as independent Pacific Island nations (including Samoa, Tonga, Palau, the Marshall Islands, and the Federated States of Micronesia). The cohort of patients in the current study includes those cared for by this practice and all patients referred to this center during this timeframe. All data were deidentified prior to use in the study and thus were exempted from patient consent requirements. This study was approved by the Institutional Review Board of The University of Hawaii at Manoa to be in compliance with ethical regulations.

HCC was diagnosed with liver biopsy or clinical/imaging criteria. In the first decade, consistent with the previous United Network for Organ Sharing (UNOS) policy regarding transplant for HCC, patients without histologic confirmation were included if they had a history of chronic liver disease, a mass of at least 2 cm seen on two imaging studies (ultrasound, CT scan or MRI), and one of the following: (1) vascular blush evident on CT scan or MRI; (2) alpha-fetoprotein (AFP) > 200 ng/ml; or (3) arteriogram confirming the tumor. More recently, the diagnosis of HCC was made with imaging alone if a contrast-enhanced study (dynamic CT or MRI) showed arterial enhancement with “washout” in the venous phase, as described by the American Association for the Study of Liver Disease (AASLD) guidelines^[20,21]^.

### Data collected

**Figure 1.**
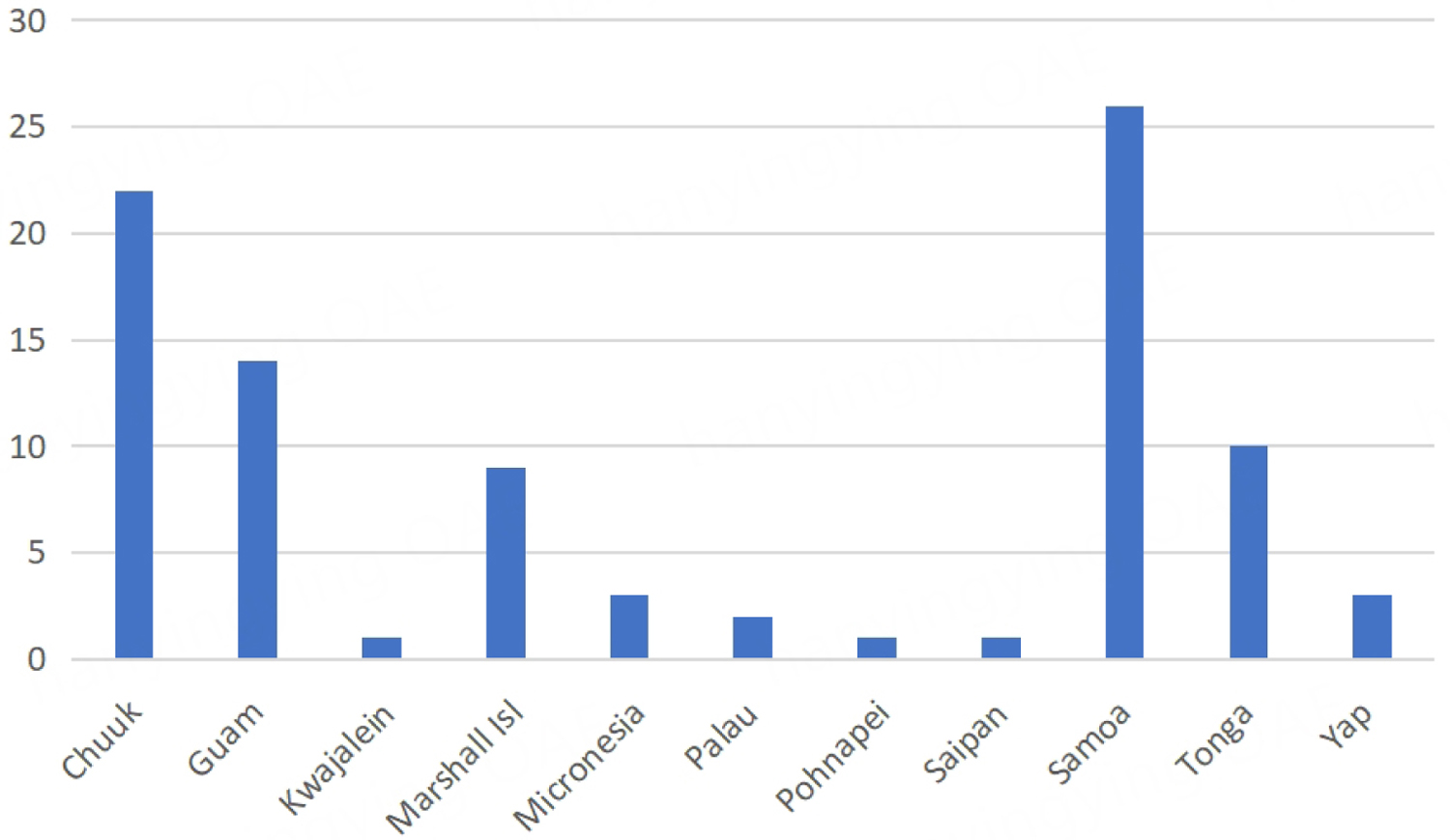
Native countries of PI-born patients.

Patient information was obtained from clinical records. Information collected included patient demographics, medical history, laboratory data, tumor characteristics, treatment, and survival. Demographic data included age, sex, birthplace, and self-reported ethnicity. Ethnicity was categorized as “White”, “Asian” (including Filipinos), “Pacific Islander” (including Native Hawaiians), “Mixed”, or “Other”. For this study, we identified 252 patients of Pacific Islander ethnicity, 153 of whom were born in the United States, 92 of whom were born on a Pacific Island other than Hawaii [Figure 1], and 7 patients whose birthplace was unknown. Patients with unknown birthplace were excluded from the analysis.

We collected information on the type of medical insurance and categorized them into “Government” insurance (Medicare, Medicaid, Veterans Association), Private insurance, or uninsured. We recorded how many years of formal education and categorized this into “finished high school” (at least 13 years) *vs*. not completing high school (less than 13 years). Patients’ zip codes were entered into the United States Census Bureau QuickFacts data access tool^[22]^ and population size was recorded. Patients were then categorized into whether they lived in an “urban” area (population ≥ 50,000) *vs*. not urban (population < 50,000).

Medical history recorded included diabetes mellitus, hyperlipidemia, previous or current malignancy other than HCC, tobacco use, and risk factors for HCC including viral hepatitis, significant alcohol use (defined as > 2 alcoholic beverages daily for ≥ 10 years), and other chronic liver diseases. In patients with a history of hepatitis C (HCV) or positive HCV serology, a complete social history was recorded and risk factors were separated into “high-risk behavior” (illicit drug use, including intravenous drug use, cocaine, methamphetamine; and incarceration) or “no high-risk behavior” (transfusions, occupational, or unknown exposure). Measured height and weight were used to determine body mass index (BMI). Obesity was defined as BMI ≥ 30. Patients were deemed to have nonalcoholic steatohepatitis (NASH) or nonalcoholic fatty liver disease (NAFLD) if they had no other known etiology for liver disease and had at least one metabolic risk factor.

The collected laboratory data included alanine aminotransferase (ALT), aspartate aminotransferase (AST), bilirubin, creatinine, albumin, prothrombin time with international normalized ratio (INR), platelet count, and AFP. Values were used to calculate a model for end-stage liver disease (MELD) score. Hepatitis B and C serologies were obtained if this information was not available from pre-existing records. For hepatitis B, a notation was made for patients with isolated hepatitis B core antibody positivity and absence of hepatitis B surface antigen. Laboratory data used for the study had been obtained within two weeks of an initial visit or at the time of the visit. We collected information on tumor size, number and major vascular invasion to determine barcelona clinic liver cancer (BCLC) stage. Tumors were also stratified to the largest tumor diameter ≥ 5 cm or < 5 cm and also tumor diameter ≥ 10 cm or < 10 cm. Normal AFP was defined as ≤ 20 ng/dL.

The process of diagnosis was noted. It was recorded whether HCC cases were diagnosed following positive screening, development of symptoms (pain, abdominal mass, weight loss, jaundice), or was an incidental finding on imaging done for unrelated reasons. Although our Liver Center recommends HCC surveillance in patients with cirrhosis with AFP and liver ultrasound every six months for patients with cirrhosis, there was no standard protocol used across the cohort. Screening methods were variable, with referring physicians using a combination of biomarkers (AFP) and/or imaging (ultrasound, CT scan or MRI) at variable intervals. HCC cases were recorded as diagnosed with “surveillance” if patients had a previous imaging study from 3–12 months prior to the current study.

### Treatments

Treatments in the cohort included liver resection, transplantation, locoregional therapies (ablation, cryosurgery, percutaneous ethanol injection, transarterial chemoembolization), or Yttrium 90 radioembolization and systemic therapies. Liver resection was considered in Child’s A patients and early Child’s B patients (Childs-Turcotte-Pugh score of 7, without any evidence of ascites or encephalopathy). Liver transplantation was considered in patients with unresectable HCC but met Milan criteria (single tumor < 5 cm or 2 to 3 tumors, each < 3 cm). All liver resections and transplantations were performed by a single surgical group.

### Statistical analysis

All analyses were performed using Excel and SPSS statistical software (IBM, version 27). Categorical variables were analyzed using ANOVA and chi-square analysis. Odds ratios with 95% confidence intervals were calculated using univariate and multivariate logistic regression. Variables significant to *P* = 0.10 in univariate analyses were included in the multivariate models along with age and sex. Variables significant at *P* < 0.05 were considered significant in the multivariate model. Overall survival was evaluated using Kaplan-Meier analysis. Predictors of survival were evaluated using Cox Proportional Hazards model, and included variables significant to *P* = 0.10 in univariate analyses as above.

### Patient demographics and comorbidities

Of 1608 HCC patients in the database, 252 were identified to be of Pacific Islander ethnicity. 7 patients were excluded from analyses , as birthplace information was not available. Of the 245 patients identified who were of Pacific Islander ethnicity, 92 patients were born in the Pacific Island countries and territories and 153 patients were born in the United States. Pacific Island-born (PI-born) patients presented at an earlier age compared to the US-born group, with mean ages of 57.3 and 62.8 years, respectively (*P* = 0.002). Both groups had a male predominance, but this was more prominent in the PI-born group. PI-born patients more frequently lived in urban areas (defined by the United States Census to be a geographic area with a population ≥ 50,000) and had a significantly higher percentage of patients who were uninsured or had government insurance compared with US-born patients (78% *vs*. 67%). PI-born patients were also less likely to be current smokers or have a smoking history. In terms of other social risk factors for HCC, there were no statistically significant differences between the groups on receiving at least a high school level education or ongoing alcohol use [Table 1].

In terms of risk factors, the PI-born group had a 14-fold higher prevalence of HBV compared to the US-born group. In contrast, HCV was less prevalent in the PI-born group than in the US-born group, with US-born patients having a nearly 4-fold higher prevalence compared to the PI-born group. Among patients with HCV, 9 of 21 (43%) PI-born patients had a social history significant for high-risk behavior compared to 61 of 80 (76%) US-born patients (OR 4.35, 95%CI: 1.56–12.50). Metabolic disorder-related liver disease, such as NASH/NAFLD, was also found to be less prevalent in PI-born patients, with 4% of patients affected compared to 17% of US-born patients. In addition, PI-born patients also had a significantly lower prevalence of other concomitant cancers compared to US-born patients, 2% *vs*. 12%, respectively [Table 2].

### Tumor characteristics/Laboratory studies

There were no differences between the groups with regard to the mean size of primary tumor, categories of largest tumor size, presence of multiple tumors, or presence of bilateral tumors; however, cirrhosis was more prevalent on presentation in the PI-born group compared to the US-born group [Table 3]. The PI-born group had lower albumin compared to the US-born group, but in all other laboratory studies, MELD score and AFP were not different between groups [Table 4].

### Diagnosis, treatments, and outcomes

Of 190 patients with screenable conditions (including cirrhosis, chronic HBV, chronic HCV), fewer PI-born patients were diagnosed through screening, with only 18% (15 of 85) of patients diagnosed on screening compared to 28% (29 of 105) of US-born patients.

**Figure 2.**
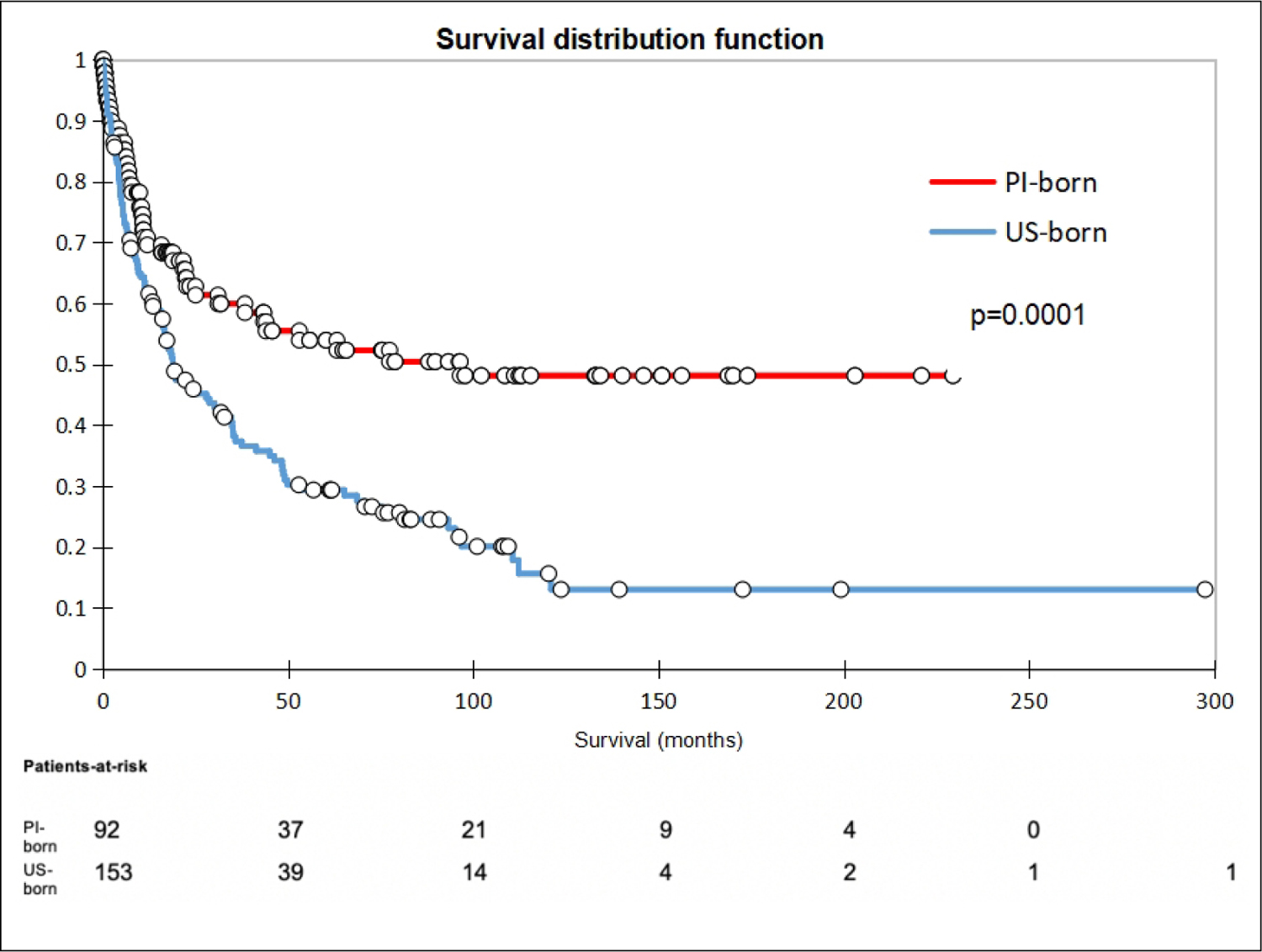
Kaplan-Meier survival curve comparing PI-born and US-born HCC patients.

**Figure 3.**
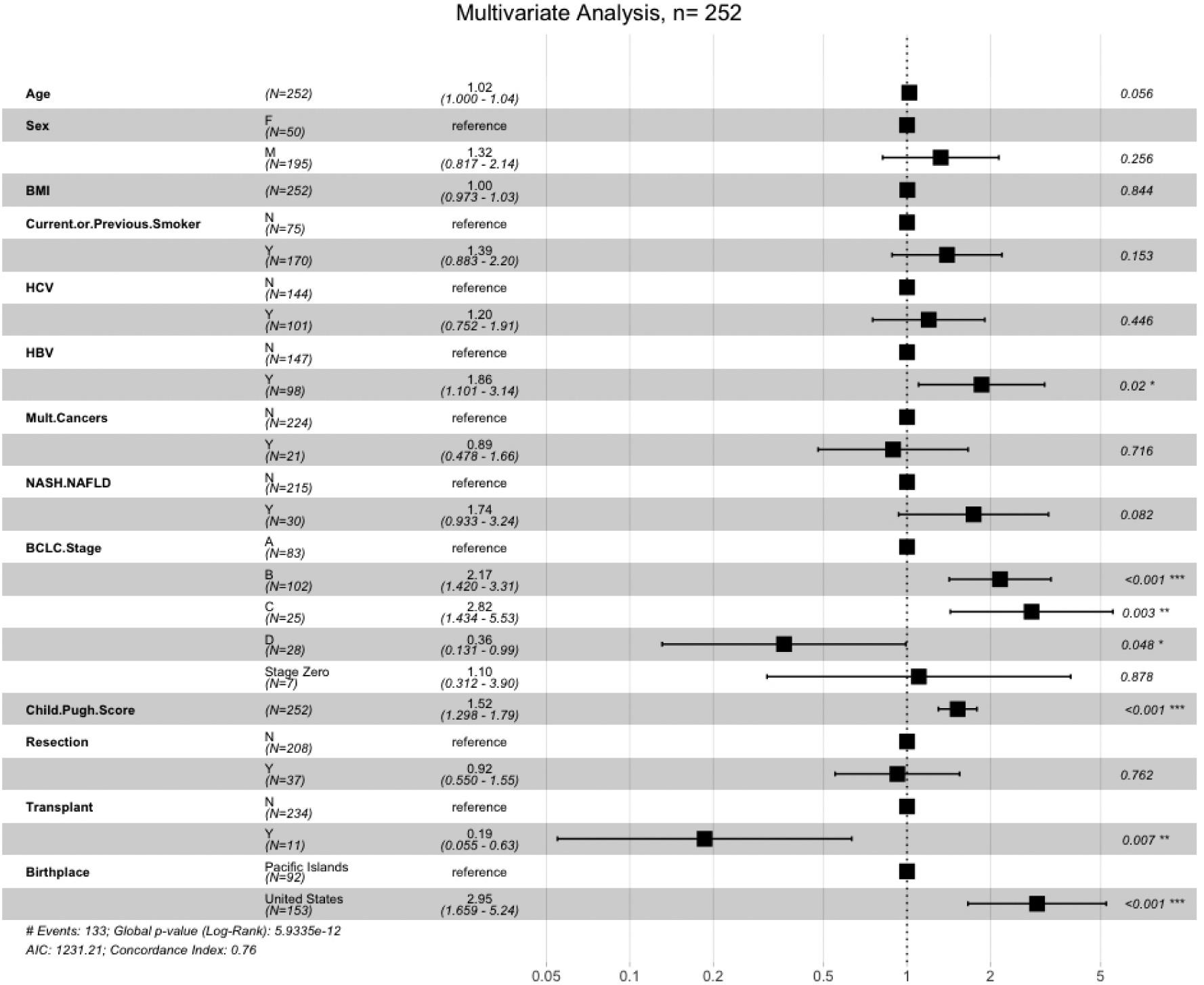
Multivariate cox regression.

There was no difference between the groups receiving surgical intervention, defined as either transplant or resection (OR 0.81, 95%CI: 0.42–1.58). Seven PI-born patients (8%) received transplants, whereas four US-born patients (3%) received transplants (OR 3.03, 95%CI: 0.86–10.64). With regard to liver resection, nine PI-born patients (10%) underwent resection compared to 28 US-born patients (19%; OR 0.49, 95%CI: 0.22–1.10). In terms of non-surgical treatment modalities, PI-born patients were less likely to receive locoregional therapy and more likely not to receive any treatment compared to US-born patients. There were no differences in the rates of undergoing ablative therapy, chemotherapy, radiation, or palliative procedures (surgical hemostasis and biliary stenting) between groups. PI-born patients were also found to have a significantly higher rate of loss to follow-up compared to US-born patients, with 11% of PI-born patients lost to follow-up compared to 1% of US-born (OR 9.21). While 1-year survival was similar in both groups, PI-born patients had better 3- and 5-year survival [Table 5]. Furthermore, median and overall survival was higher in PI-born patients compared to US-born patients (*P* = 0.0001, Figure 2). After adjustment for age, sex, BMI, BCLC stage, Child-Pugh score, HCC risk factors, history of smoking, and previous non-HCC malignancy, Cox regression demonstrated that US-born status was associated with higher mortality risk, and liver transplant was associated with lower mortality risk [Figure 3].

## DISCUSSION

Chronic HBV remains one of the primary risk factors for HCC and is responsible for at least 50% of cases of HCC worldwide^[23]^. In the United States, Asian Americans and Pacific Islanders account for more than 50% of chronic HBV cases, with most cases occurring in immigrants^[24,25]^. HBV prevalence remains high in Pacific Island countries and territories and is estimated at 5%−7%^[14]^. Universal HBV vaccination programs have been introduced to many of these countries; however, limited vaccine availability and obstacles to vaccine delivery (vaccine storage, limited antenatal screening, home births, and cost) have limited their success^[14,26–28]^. While HBV is mostly acquired through vertical transmission in Pacific Island countries^[29]^, HBV prevalence increases until the last two decades of life, suggesting multiple modes of transmission^[14]^. The endemicity of HBV in this population was further validated in the current study, with PI-born patients found to have a 14 times higher likelihood of having HBV compared to US-born patients.

Disparities in the treatment of chronic HBV infections further perpetuate the high burden of disease in these populations. Studies on treatment with antivirals have demonstrated a reduction in the risk of cirrhosis and short- and medium-term risk of development of HCC^[28,30,31]^. However, medication costs and limited resources for drug delivery/monitoring present major obstacles to treatment in resource-constrained areas^[32]^. Without HBV treatment, these patients are predisposed to increased progression to cirrhosis and HCC. This is evident in our study, as PI-born patients were younger on presentation and were twice as likely to have cirrhosis compared with US-born patients.

While HCV is the major viral hepatitis in the United States, there is a relatively low prevalence of HCV (2.6%) in the Pacific Islands which may be attributed to their geographic isolation with low rates of immigration from high prevalence areas^[14,33]^ and generally low rates of injection drug use^[34,35]^. In our study, US-born patients had a 4-fold higher likelihood of HCV in comparison to PI-born patients. Our study also corroborates what has been reported previously, as the US-born Pacific Islanders with HCV were significantly more likely to engage in high-risk behaviors associated with HCV compared to PI-born patients.

While the true prevalence of NAFLD and risk of progression to HCC PI-born Pacific Islanders remains unclear, an estimated 50%−90% are classified as overweight or obese and 15%−45% suffer from type 2 diabetes in some regions^[14,36]^. In the United States, these patterns persist, with Native Hawaiians/Pacific Islanders reported being 80% more likely to be obese than non-Hispanic whites, making them one of the highest risk groups for developing metabolic syndrome, type 2 diabetes, and cardiovascular disease^[17,37]^. NAFLD is a significantly more common cause of cirrhosis in Native Hawaiians (31.5%) compared to whites (21.7%)^[38]^. While there was no difference in the prevalence of obesity, diabetes, or hyperlipidemia between PI-born and US-born in our study, the US-born patients had nearly a 5 times higher likelihood of having NASH/NAFLD compared to PI-born patients. This may be suggestive of a genetic predisposition to the development of NASH/NAFLD in Pacific Islanders, and furthermore, the difference observed between US-born and PI-born cohorts may reflect an epigenetic component and an impact of adoption of a Western diet and lifestyle, but this would need to be explored further^[39,40]^.

Tobacco and alcohol use are also significant risk factors that promote HCC carcinogenesis. Tobacco use remains a common practice among Pacific Islanders; in Pacific Island countries and territories, reported prevalence ranges from 22%−70% depending on region^[41,42]^, compared to the reported 18.7% of Native Hawaiian/Pacific Islanders in the United States smoking in the previous year^[43]^. In this study, US-born patients were 4 times more likely to be current smokers and twice as likely to have smoked in the past compared to PI-born patients. Alcohol misuse, though historically uncommon, has also become an important risk factor in PI-born populations, especially with growing urbanization and poverty^[14,44,45]^. Our study suggests that significant alcohol use among PI-born HCC patients was similar to that of US-born patients.

Socioeconomic factors including financial status, medical insurance, health literacy and language barriers may all affect access to care, especially among the newly immigrated^[13,15,46]^. These factors may affect the early detection of HCC and the opportunity for curative therapy with liver resection or transplant. While this study cannot delineate the specifics of access to care, PI-born patients were twice as likely to live in an urban area, and twice as likely to have government health insurance or be uninsured compared to US-born patients. In addition, PI-born patients were less likely to have had HCC diagnosed on surveillance, 3 times as likely to forego treatment, and 9 times more likely to be lost to follow-up compared to US-born patients. This suggests that despite the availability of care in urban areas, PI-born Pacific Islander patients face challenges in access to medical care, such as uninsured/underinsured status, language barriers, or lower health literacy. Differences in seeking care may also reflect differences in the prevalence of cultural beliefs or practices that conflict with Western ideas of health and healthcare between PI and US-born groups^[47]^. However, despite these barriers, PI-born patients were equally likely to receive curative surgical intervention as US-born patients. Mortality risk was positively associated with US-born status and negatively associated with undergoing transplantation. While both groups had comparable survival at 1 year, PI-born patients were observed to have higher long-term survival compared to US-born patients, which may be partially attributable to the higher number of patients receiving liver transplants in the PI-born group. The observed difference in the number of transplants received between the groups is likely multifactorial. For transplantation, our center does not consider candidates with active substance abuse, smoking, severe cardiopulmonary disease or untreated non-HCC malignancies. While some of these exclusions were not captured in this study, patients in the US-born group were more likely to be active smokers, have other non-HCC malignancies, and may include more patients with active substance use which may have precluded transplantation. Beyond transplantation, other factors such as etiology of HCC may help to explain the observed difference in survival between groups. Previous studies have demonstrated poorer overall and recurrence-free survival after transplantation in patients with HCV-associated HCC^[48]^. This may have contributed to poorer survival in the US-born cohort of the current study due to the higher rates of HCV in this group; however, more research and larger sample sizes are needed to draw accurate conclusions in this population.

A limitation of the study is that it is a retrospective observational study using data collected over three decades from a single center. As such, there is the potential for bias and confounding associated with the observational study design. Furthermore, much may have changed in the management of HCC over this long period of time. Improvements in imaging and referral patterns may likely have affected earlier detection, surveillance, and treatment in more recent years. Another potential limitation of the study is that information on the length of time since immigration was not recorded in the Pacific Island-born group, so any temporal effect of immigration was not evaluated. Additionally, as Pacific Island-born patients who have moved to the United States may differ from those who remain living in the Pacific Islands, these results cannot be generalized to all Pacific Island-born patients.

Despite these limitations, our study has much granular detail about underlying liver disease and comorbidities, which are not available from large administrative databases. This is a very large series of HCC in Pacific Islanders, one of the fastest growing ethnic groups in the United States that suffers from a high burden of liver disease. We found that HBV continues to be the primary risk factor for disease in PI-born patients, while exposures and behaviors associated with a Westernized lifestyle are significant risk factors in US-born patients. We demonstrate that despite the perceived hardships of recent immigration, our PI-born patients were able to navigate the health system and have an equal opportunity for curative therapy with liver resection or transplant as US-born patients.

## Figures and Tables

**Table 1.** Demographics of HCC patients of Pacific Island ancestry

	PI-born (*n* = 92)	US-born (*n* = 153)	*P* value	Unadjusted OR, 95%CI
Age (years)	57.3 ( 11.4)	61.8 ( 10.0)	***P* = 0.002**	
Sex: male	80 (87%)	115 (75%)		2.20 (**1.08–4.48)**
Government insurance or uninsured	74 (78%)	100 (67%)		2.06 (**1.10–3.82)**
Completed high school	47 (65%)	97 (76%)		0.58 (0.31–1.10)
Urban	48 (52%)	49 (32%)		2.27 (**1.33–3.87)**

HCC: Hepatocellular carcinoma; PI-born: Pacific Island-born.

**Table 2.** Risk factors and comorbidities of Pacific Island patients with HCC

	PI-born (*n* = 92)	US-born (*n* = 153)	Unadjusted OR, 95%CI
Hepatitis B	56 (61%)	17 (11%)	**14.10** (**7.50–26.50)**
Hepatitis C	21 (23%)	80 (52%)	**0.27** (**0.15–0.48)**
NASH/NAFLD	4 (4.4%)	26 (17%)	**0.22** (**0.07–0.65)**
Other cancer	2 (2.2%)	19 (12%)	**0.16 (0.04–0.69)**
Diabetes mellitus	35 (38%)	56 (37%)	1.16 (0.62–1.81)
Hypertension	47 (55%)	91 (66%)	0.65 (0.38–1.13)
Hyperlipidemia	28 (31%)	45 (31%)	1.01 (0.57–1.78)
BMI ≥ 30	46 (56%)	66 (46%)	1.47 (0.85–2.54)
Smoking (current)	7 (13%)	39 (37%)	**0.25 (0.10–0.60)**
Smoking history	54 (59%)	116 (77%)	**0.44** (**0.25–0.77)**
Alcohol use	53 (58%)	95 (62%)	0.85 (0.50–1.44)
Hepatitis C + history of social risk factors	9 (43%)	61 (76%)	**4.35 (1.56–12.50)**

HCC: Hepatocellular carcinoma; PI-born: Pacific Island-born; NASH: nonalcoholic steatohepatitis; NAFLD: nonalcoholic fatty liver disease.

**Table 3.** Tumor characteristics of Pacific Island patients with HCC

		PI-born (*n* = 92)	US-born (*n* = 153)	*P* value	Unadjusted OR, 95%CI
Mean size of primary tumor (cm)		6.49 ( 5.01)	6.58 ( 4.84)	*P* = 0.90	
Size ≥ 5cm		44 (48%)	79 (52%)	*P* = 0.58	0.86 (0.51–2.11)
Size ≥ 10cm		25 (27%)	37 (24%)	*P* = 0.61	1.17 (0.65–2.11)
Multiple tumors		37 (40%)	52 (34%)	*P* = 0.33	1.31 (0.77–2.23)
Bilateral tumors		14 (18%)	24 (17%)	*P* = 0.92	1.04 (0.50–2.15)
Met Milan criteria		38 (41%)	65 (42%)	*P* = 0.78	0.95 (0.56–1.61)
Cirrhosis		76 (84%)	107 (70%)	***P*** = **0.02**	**2.28 (1.17–4.45)**
BCLC stage	0	1 (1%)	6 (4%)	*P* = 0.22	0.26 (0.03–2.24)
	A	30 (33%)	53 (35%)	*P* = 0.70	0.89 (0.52–1.55)
	B	36 (39%)	64 (42%)	*P* = 0.63	0.87 (0.52–1.48)
	C	11 (12%)	14 (9%)	*P* = 0.52	1.33 (0.57–3.07)
	D	14 (15%)	14 (9%)	*P* = 0.16	1.76 (0.80–3.87)

HCC: Hepatocellular carcinoma; PI-born: Pacific Island-born; BCLC: barcelona clinic liver cancer.

**Table 4.** Clinical laboratory analytes of Pacific Island patients with HCC

	PI-born (*n* = 92)	US-born (*n* = 153)	*P* value	Unadjusted OR, 95%CI
Albumin (mg/dL)	3.24 ( 0.77)	3.54 ( 0.65)	***P* = 0.003**	
Bilirubin (mg/dL)	2.42 ( 4.30)	2.14 ( 4.11)	*P* = 0.62	
Creatinine (mg/dL)	1.26 ( 0.97)	1.09 ( 0.71)	*P* = 0.14	
Platelets (x10^3^/μl)	178 ( 101)	179 ( 90)	*P* = 0.98	
INR	1.24 ( 0.28)	1.18 ( 0.22)	*P* = 0.06	
Median AFP (ng/dL	25.9	34.8	*P* = 0.90	
Normal AFP (< 20 ng/dL)	41 (45%)	56 (38%)		1.33 (0.79–2.26)
Mean MELD score	12 ( 5)	11 ( 5)	*P* = 0.09	

HCC: Hepatocellular carcinoma; PI-born: Pacific Island-born; NASH: nonalcoholic steatohepatitis; NAFLD: nonalcoholic fatty liver disease; BCLC: barcelona clinic liver cancer; INR: international normalized ratio; AFP: alpha-fetoprotein; MELD: model for end-stage liver disease.

**Table 5.** Diagnosis, treatment, and outcomes

	PI-born (*n* = 92)	US-born (*n* = 153)	Unadjusted OR, 95%CI
Diagnosis on screening	15 (18%)	29 (28%)	0.57, 0.28–1.15
Locoregional therapy	23 (27%)	75 (51%)	0.36, **0.20–0.63**
Ablation	7 (8%)	16 (10%)	0.70, 0.28–1.78
Chemotherapy	2 (2%)	2 (1%)	1.68, 0.23–12.11
Radiation	1 (1%)	1 (1%)	1.67, 0.10–27.03
Surgical intervention (transplant or resection)	16 (18%)	28 (22%)	0.81, 0.42–1.58
Transplant	7 (8%)	4 (3%)	3.03, 0.86–10.64
Resection	9 (10%)	24 (19%)	0.49, 0.22–1.10
Palliative (surgical hemostasis, biliary stent)	3 (3%)	0	12.00, 0.61–235.11
No treatment	40 (43%)	31 (20%)	3.22, **1.77–5.87**
Loss to follow-up	10 (11%)	2 (1%)	9.21, **1.97–43.03**
1-year survival	51 (62%)	89 (59%)	1.15, 0.66–1.99
3-year survival	36 (44%)	45 (30%)	1.84, **1.05–3.22**
5-year survival	31 (38%)	33 (22%)	2.17, **1.20–3.92**
Median survival (months)	22.4	16.9	

PI-born: Pacific Island-born.

## Data Availability

Data inquiries may be forwarded to the corresponding author.

## References

[1] National Cancer Institute, NIH. Liver cancer causes, risk factors, and prevention. Available from https://www.cancer.gov/types/liver/what-is-liver-cancer/causes-risk-factors [Last accessed 15 Mar 2023].

[2] Xie Y Hepatitis B virus-associated hepatocellular carcinoma. Adv Exp Med Biol 2017;1018:11–21. 29052129 10.1007/978-981-10-5765-6_2

[3] Younossi ZM. Non-alcoholic fatty liver disease - a global public health perspective. J Hepatol 2019;70:531–44. 30414863 10.1016/j.jhep.2018.10.033

[4] Estes C, Razavi H, Loomba R, Younossi Z, Sanyal AJ. Modeling the epidemic of nonalcoholic fatty liver disease demonstrates an exponential increase in burden of disease. Hepatology 2018;67:123–33. 28802062 10.1002/hep.29466 PMC5767767

[5] Wong LL, Hernandez B, Kwee S, Albright CL, Okimoto G, Tsai N. Healthcare disparities in Asians and Pacific Islanders with hepatocellular cancer. Am J Surg 2012;203:726–32. 22227170 10.1016/j.amjsurg.2011.06.055 PMC3524307

[6] Yang B, Liu JB, So SK, Disparities in hepatocellular carcinoma incidence by race/ethnicity and geographic area in California: implications for prevention. Cancer 2018;124:3551–9. 30113700 10.1002/cncr.31598 PMC6436543

[7] Danos D, Leonardi C, Gilliland A, Increased risk of hepatocellular carcinoma associated with neighborhood concentrated disadvantage. Front Oncol 2018;8:375. 30254987 10.3389/fonc.2018.00375 PMC6141716

[8] Shebl FM, Capo-Ramos DE, Graubard BI, McGlynn KA, Altekruse SF. Socioeconomic status and hepatocellular carcinoma in the United States. Cancer Epidemiol Biomarkers Prev 2012;21:1330–5. 22669949 10.1158/1055-9965.EPI-12-0124 PMC3647693

[9] Stewart SL, Kwong SL, Bowlus CL, Racial/ethnic disparities in hepatocellular carcinoma treatment and survival in California, 1988–2012. World J Gastroenterol 2016;22:8584–95. 27784971 10.3748/wjg.v22.i38.8584 PMC5064040

[10] Chang ET, Keegan TH, Gomez SL, The burden of liver cancer in Asians and Pacific Islanders in the Greater San Francisco Bay Area, 1990 through 2004. Cancer 2007;109:2100–8. 17385214 10.1002/cncr.22642 PMC2777532

[11] McGlynn KA, Petrick JL, El-Serag HB. Epidemiology of hepatocellular carcinoma. Hepatology 2021;73 Suppl 1:4–13. 10.1002/hep.31288 PMC7577946 32319693

[12] Harlan LC, Parsons HM, Wiggins CL, Stevens JL, Patt YZ. Treatment of hepatocellular carcinoma in the community: disparities in standard therapy. Liver Cancer 2015;4:70–83. 26020030 10.1159/000367729 PMC4439839

[13] Mathur AK, Osborne NH, Lynch RJ, Ghaferi AA, Dimick JB, Sonnenday CJ. Racial/ethnic disparities in access to care and survival for patients with early-stage hepatocellular carcinoma. Arch Surg 2010;145:1158–63. 21173289 10.1001/archsurg.2010.272

[14] Howell J, Van Gemert C, Lemoine M, Thursz M, Hellard M. Overview of hepatitis B prevalence, prevention, and management in the Pacific Islands and Territories. J Gastroenterol Hepatol 2014;29:1854–66. 25131570 10.1111/jgh.12684

[15] National Academies of Sciences, Engineering, and Medicine; Health and Medicine Division; Board on Health Care Services; Committee on Health Care Utilization and Adults with Disabilities. Factors that affect health-care utilization. In: Health-care utilization as a proxy in disability determination. Washington (DC): National Academies Press (US); 2018. p. 21–38. 29782136

[16] U.S. Census Bureau. New census data show differences between urban and rural populations. Available from: https://content.govdelivery.com/accounts/USCENSUS/bulletins/1782904 [Last accessed on 14 Mar 2023].

[17] U.S. Department of Health and Human Services, Office of Minority Health. Minority population profiles: Native Hawaiian/Other Pacific Islander. Available from: https://minorityhealth.hhs.gov/omh/browse.aspx?lvl=3&lvlid=65 [Last accessed on 14 Mar 2023].

[18] Kaiser Family Foundation (KFF). Health care disparities among Asian, Native Hawaiian, and Other Pacific Islander (NHOPI) People. Available from: https://www.kff.org/racial-equity-and-health-policy/issue-brief/health-care-disparities-among-asian-native-hawaiian-and-other-pacific-islander-nhopi-people/ [Last accessed on 14 Mar 2023].

[19] NIH. State cancer profiles: liver and bile duct, 2014–2018. Available from: www.statecancerprofiles.cancer.gov [Last accessed on 14 Mar 2023].

[20] Heimbach JK, Kulik LM, Finn RS AASLD guidelines for the treatment of hepatocellular carcinoma. Hepatology 2018;67:358–80. 28130846 10.1002/hep.29086

[21] Roberts LR, Sirlin CB, Zaiem F, Imaging for the diagnosis of hepatocellular carcinoma: a systematic review and meta-analysis. Hepatology 2018;67:401–21. 28859233 10.1002/hep.29487

[22] U.S. Census Bureau. Population estimates, July 1, 2021 (V2021); quick facts. Available from https://www.supremecourt.gov/opinions/URLs_Cited/OT2021/19-1392/19-1392-7.pdf [Last accessed on 14 Mar 2023].

[23] Xie Y Hepatitis b virus-associated hepatocellular carcinoma. In: Cai Q, Yuan Z, Lan K. (eds) Infectious agents associated cancers: epidemiology and molecular biology. advances in experimental medicine and biology, vol 1018. Singapore: Springer; 2017. p. 11–21. 29052129 10.1007/978-981-10-5765-6_2

[24] CDC. People born outside of the United States and viral hepatitis. Available from: https://www.cdc.gov/hepatitis/populations/Born-Outside-United-States.htm [Last accessed on 14 Mar 2023].

[25] U.S. Department of Health and Human Services; National Institute of Diabetes and Digestive and Kidney Diseases. Hepatitis B. Available from: https://www.niddk.nih.gov/health-information/liver-disease/viral-hepatitis/hepatitis-b [Last accessed on 14 Mar 2023].

[26] Lasitani S, Hattori C, Elisara T, Araneta MR. Assessing hepatitis B knowledge among Native Hawaiians and Pacific Islanders in San Diego. J Immigr Minor Health 2021;23:1193–7. 34255232 10.1007/s10903-021-01236-1 PMC8599383

[27] Abara WE, Collier MG, Teshale EH. Impact of universal infant hepatitis B vaccination in the US-affiliated Pacific Islands, 1985–2015. Vaccine 2017;35:997–1000. 28117171 10.1016/j.vaccine.2017.01.020 PMC10168596

[28] Thursz M, Njie R, Lemoine M. Global eradication of hepatitis B-feasible or fallacy? Nat Rev Gastroenterol Hepato 2012;9:492–4. 10.1038/nrgastro.2012.155 22868658

[29] Mast EE, Alter MJ, Margolis HS. Strategies to prevent and control hepatitis B and C virus infections: a global perspective. Vaccine 1999;17:1730–3. 10194830 10.1016/s0264-410x(98)00415-0

[30] Papatheodoridis GV, Lampertico P, Manolakopoulos S, Lok A. Incidence of hepatocellular carcinoma in chronic hepatitis B patients receiving nucleos(t)ide therapy: a systematic review. J Hepatol 2010;53:348–56. 20483498 10.1016/j.jhep.2010.02.035

[31] Wong RJ, Jain MK, Therapondos G, Niu B, Kshirsagar O, Thamer M. Antiviral therapy reduces risk of cirrhosis in noncirrhotic HBV patients among 4 urban safety-net health systems. Am J Gastroenterol 2021;116:1465–75. 33661148 10.14309/ajg.0000000000001195

[32] Thursz M, Cooke GS, Hall AJ. Hepatitis B treatment in resource poor settings: time for action. Trop Med Int Health 2010;15:2–4. 19843297 10.1111/j.1365-3156.2009.02410.x

[33] Hanafiah K, Groeger J, Flaxman AD, Wiersma ST. Global epidemiology of hepatitis C virus infection: new estimates of age-specific antibody to HCV seroprevalence. Hepatology 2013;57:1333–42. 23172780 10.1002/hep.26141

[34] Howard J, Ali H. Injecting drug use among young people in Pacific Island countries and territories: a review of the evidence. Drug Alcohol Rev 2013;32:631–3. 24102862 10.1111/dar.12071

[35] Ali H, Howard J. Prevalence of injecting drug use among youth in the Pacific Island countries and territories: what is the evidence? Asia Pac J Public Health 2011;23:112–4. 21169605 10.1177/1010539510390663

[36] Islanders pay heavy price for abandoning traditional diet. Bull World Health Organ 2010;88:484–5. 20616964 10.2471/BLT.10.010710 PMC2897991

[37] Pattison RJ, Esteban JP, Sempokuya T, Nonalcoholic fatty liver disease: an important consideration for primary care providers in Hawai’i. Hawaii J Health Soc Welf 2020;79:180–6. 32524096 PMC7281344

[38] Setiawan VW, Stram DO, Porcel J, Lu SC, Le Marchand L, Noureddin M. Prevalence of chronic liver disease and cirrhosis by underlying cause in understudied ethnic groups: the multiethnic cohort. Hepatology 2016;64:1969–77. 27301913 10.1002/hep.28677 PMC5115980

[39] Sliz E, Sebert S, Würtz P, NAFLD risk alleles in PNPLA3, TM6SF2, GCKR and LYPLAL1 show divergent metabolic effects. Hum Mol Genet 2018;27:2214–23. 29648650 10.1093/hmg/ddy124 PMC5985737

[40] Lee J, Kim Y, Friso S, Choi SW. Epigenetics in non-alcoholic fatty liver disease. Mol Aspects Med 2017;54:78–88. 27889327 10.1016/j.mam.2016.11.008

[41] Rasanathan K, Tukuitonga CF. Tobacco smoking prevalence in Pacific Island countries and territories: a review. N Z Med J 2007;120:U2742. 17972962

[42] Kessaram T, McKenzie J, Girin N, Tobacco smoking in islands of the Pacific Region, 2001–2013. Prev Chronic Dis 2015;12:E212. 26632953 10.5888/pcd12.150155 PMC4675401

[43] CDC. Asian, Native Hawaiian, and Pacific Islander people experience a health burden from commercial tobacco. Available from: https://www.cdc.gov/tobacco/health-equity/anhpi/health-burden.html [Last accessed on 14 Mar 2023].

[44] Kessaram T, McKenzie J, Girin N, Alcohol use in the Pacific region: results from the STEPwise approach to surveillance, Global School-Based Student Health Survey and Youth Risk Behavior Surveillance System. Drug Alcohol Rev 2016;35:412–23. 26358376 10.1111/dar.12328 PMC5049666

[45] Subica AM, Guerrero E, Aitaoto N, Moss HB, Iwamoto D, Wu LT. Hazardous drinking, alcohol use disorders, and need for treatment among Pacific Islander young adults. Am J Orthopsychiatry 2020;90:557–66. 32352815 10.1037/ort0000456 PMC9048751

[46] Floyd A, Sakellariou D. Healthcare access for refugee women with limited literacy: layers of disadvantage. Int J Equity Health 2017;16:195. 29126420 10.1186/s12939-017-0694-8 PMC5681803

[47] Morisako AK, Tauali’i M, Ambrose AJH, Withy K. Beyond the ability to pay: the health status of Native Hawaiians and Other Pacific Islanders in relationship to health insurance. Hawaii J Med Public Health 2017;76 (3 Suppl 1):36–41. 28435757 PMC5375012

[48] Shindoh J, Hashimoto M, Watanabe G. Surgical approach for hepatitis C virus-related hepatocellular carcinoma. World J Hepatol 2015;7:70–77. 25624998 10.4254/wjh.v7.i1.70 PMC4295196

